# Nutritional Gender-Specific Differences in Head and Neck Cancer Patients Treated with (Chemo)Radiotherapy: Results from a Prospective Trial

**DOI:** 10.3390/cancers16234080

**Published:** 2024-12-05

**Authors:** Rouzbeh Zohri, Lorenz Hahn, Niloufar Seyedi, Cordula Petersen, Christian Ziemann, Jakob Abel, Laura Magdalena Kutz, Andreas Krüll, Charlotte Flüh, Carolin Ehresmann, Oksana Zemskova, Larysa Liubich, Dirk Rades, Anastassia Löser

**Affiliations:** 1 Department of Osteology, Outpatient Center, University Medical Center Hamburg-Eppendorf, 22529 Hamburg, Germany; 2 Department of Trauma Surgery, Orthopaedics and Sports Traumatology, BG Clinic Hamburg, 21033 Hamburg, Germany; 3 Department of Trauma Surgery and Orthopaedics, University Medical Center Hamburg-Eppendorf, 20246 Hamburg, Germany; 4 Tetra Pak Processing GmbH, 21509 Reinbek, Germany; 5 Department of Stem Cell Transplantation, University Medical Center Hamburg-Eppendorf, 20251 Hamburg, Germany; 6 Department of Oncology, Hematology and Bone Marrow Transplantation with the Section Pneumology, University Medical Center Hamburg-Eppendorf, 20251 Hamburg, Germany; 7 Department of Radiotherapy and Radiation Oncology, University Medical Center Hamburg-Eppendorf, 20251 Hamburg, Germany; 8 Department of Radiotherapy, Outpatient Center, University Medical Center Hamburg-Eppendorf, 20251 Hamburg, Germany; 9 Department of Radiotherapy, University Medical Center Schleswig-Holstein, Campus Lübeck, 23562 Lübeck, Germany; 10 Department of Otorhinolaryngology, Head and Neck Surgery, Charité-Universitätsmedizin Berlin, 10117 Berlin, Germany; 11 Department of Neurosurgery, University Medical Center Göttingen, 37075 Göttingen, Germany; 12 State Institution Romodanov Neurosurgery Institute, National Academy of Medical Sciences of Ukraine, 04112 Kyiv, Ukraine; 13 Section for Translational Surgical Oncology and Biobanking, Department of Surgery, University of Lübeck, University Medical Center Schleswig-Holstein/Lübeck, 23562 Lübeck, Germany

**Keywords:** Malnutrition, fat-free mass index, bioelectrical impedance analysis, phase angle, survival

## Abstract

**Background/Objectives**: This analysis aims to evaluate gender-specific differences in nutritional status, body weight changes, and their impact on overall survival (OS) in head and neck cancer (HNC) patients undergoing (chemo)radiotherapy (CRT). **Methods**: Between 2018 and 2020, 61 HNC (17 female and 44 male) patients were prospectively recruited to receive curative (chemo)radiotherapy. Nutritional assessments included dietary questionnaire screenings and records, anthropometric methods (body mass index, BMI, body composition via bioelectrical impedance analysis (BIA)), and the determination of biomarkers like albumin and CRP. Assessments were conducted before, during, and after (chemo)radiotherapy. **Results**: Gender differences were observed at baseline in Karnofsky performance status (*p* = 0.01), daily calorie intake (*p* = 0.04), phase angle (PA) (*p* = 0.003), and fat-free mass index (FFMI) (*p* < 0.001). During CRT, males showed a larger increase in calorie deficit (*p* < 0.001) and greater reductions in BMI, FFMI, and PA compared to females. Malnutrition risk (MUST score) increased significantly in males (*p* = 0.008) but not in females. Albumin and total protein declined in both genders, with a more pronounced drop in albumin for females. Survival analysis revealed that, for males, several factors, including baseline calorie deficit, BMI, PA, and FFMI, were linked to survival. For females, only albumin at therapy end was significantly associated with survival (*p* < 0.001). In multivariable analysis, baseline PA remained a significant predictor of survival for males (*p* = 0.026). **Conclusions**: Our findings suggest distinct gender differences in the nutritional and biochemical responses of HNC patients undergoing CRT, indicating the importance of tailored, gender-specific nutritional support during treatment.

## Simple Summary

Head and neck cancer (HNC) patients often experience serious side effects from treatments like chemotherapy and radiotherapy, which can lead to malnutrition and impact their overall health status. Men and women may face different nutritional challenges during these treatments, potentially affecting their treatment outcomes. This study investigates the gender-specific nutritional status of HNC patients over the course of their treatment, examining differences in body weight, body composition, and their impact on survival. While we found significant declines in albumin and total protein levels in both genders, females exhibited a more notable decrease in albumin levels. Survival analysis revealed that for males, the phase angle, an indicator of cellular health, was linked to survival. For females, albumin levels at the end of treatment were significant predictors of survival.

## 1. Introduction

Squamous cell carcinoma of the head and neck (HNSCC) encompasses a diverse group of malignancies affecting the oral cavity, pharynx, and larynx. Standard treatment options for HNSCC include surgery, radiotherapy (RT), chemotherapy, or a combination of these modalities, with chemoradiotherapy (CRT) being a cornerstone of therapy, especially for advanced stages. While CRT constitutes a highly effective treatment approach, it may affect the patients’ nutritional status due to mucositis, dysphagia, or xerostomia. These side effects may lead to substantial weight loss and malnutrition, which, in turn, negatively impact overall survival (OS) [1,2].

Being predominantly diagnosed in men [3], there is a gender-specific discrepancy in the prevalence of HNC. However, the reasons for this are not yet fully understood [4]. Emerging evidence suggests that the challenges faced by HNC patients differ by gender: An analysis of “The Cancer Genome Atlas” (TCGA) dataset reported that men present more frequently with HPV (human papilloma virus)-positive tumors, while HPV-negative tumors are more common in women who face poorer OS. In addition, HPV-negative tumors were more likely to exhibit so-called BRWED3 mutations, commonly found in women [5]. Apart from molecular biological factors, there are also therapy-associated features that could have a negative impact on the survival of female HNC patients. For example, a Californian research group observed that female HNC patients were less likely to receive a dose-intensive chemo- and radiotherapy [6]. The fact that treatment decisions differ between men and women was also confirmed by Dittberner et al., who retrospectively examined 8288 HNC patients within the Thuringian Cancer Registry [7]. In contrast, other authors suggest gender disparities regarding systemic inflammatory biomarkers, such as platelet count and neutrophil-to-lymphocyte ratio (NLR) [8]. In 2020, De Courcy et al. reported gender differences regarding the reaction to RT based on the availability of DNA repair mechanisms. These authors emphasized that precision medicine requires a gender-based approach and that patients’ gender should be taken into account when developing therapy protocols [9].

Previous studies have examined the general impact of malnutrition in HNC patients [2,10]. However, data on gender-related nutritional differences during CRT are still very limited [11]. Identifying and addressing these differences could enhance the personalization of nutritional interventions, optimize treatment tolerance, and improve clinical outcomes for both male and female patients. Differences in body composition, energy requirements, and hormonal influences between men and women could contribute to varying nutritional needs and treatment responses, thus making a step forward to personalized medicine. Understanding these differences is essential, as gender-specific factors could influence the risk of treatment-related malnutrition and the need for nutritional support during and after therapy.

This analysis is based on the so-called HEADNUT (head and neck cancer patients under (chemo)radiotherapy undergoing nutritional intervention) cohort, which was initially published in 2021 [2]. However, this sub-analysis aims to evaluate gender-specific nutritional differences in HNC patients undergoing CRT. Using data from a prospective clinical trial, we assessed the variations in nutritional status, body weight changes, and their impact on OS in male and female HNC patients throughout their treatment course.

## 2. Materials and Methods

### 2.1. Study Design

This analysis is based on data from a prospective, monocentric patient cohort consisting of 61 HNC patients who either received curative radiotherapy or combined CRT within the framework of the HEADNUT trial. Patient recruitment was started in 2018 and continued until 2020 at the Department of Radiotherapy at the University Medical Center Hamburg-Eppendorf. Adult patients with histologically confirmed HNSCC were eligible for study participation. Patients with inlying pacemakers, treatment interruptions, nasopharyngeal carcinoma, or other solid tumor diseases within the last 15 years were excluded from the analysis. Written informed consent was obtained from all patients. The study was approved by the local ethics committee in Hamburg, Germany (PV5818).

Nutritional data were collected at multiple time points: before treatment initiation, every two weeks during therapy, and after treatment completion (initially after 6-8 weeks, then semiannually). Assessments included detailed dietary records, anthropometric measurements (BMI), validated nutritional screening questionnaires (MUST, NRS-2002, and Nutriscore) [12,13,14], and bioelectrical impedance analysis (BIA) to assess body composition, including fat and muscle mass. The principle of BIA has previously been explained in detail [15]. The phase angle (PA) as well as the fat-free mass index (FFMI) from the BIA were included in the present analysis. The PA acts as an indicator of cell membrane integrity and cellular health [16].

Biochemical markers, such as albumin, total protein count, and C-reactive protein (CRP) [17,18,19], were also measured to evaluate the nutritional and inflammatory status. Detailed information on sample size calculation, study design, and patient selection was previously published [2].

Patients in combined CRT received cisplatin at 100 mg/m^2^ every three weeks. For those with reduced performance status, a dose-reduced regimen of 40 mg/m^2^ weekly was used. When cisplatin was contraindicated, an alternative regimen of 5-fluorouracil (5-FU) (600 mg/m^2^ on days 1–5) with Mitomycin C (10 mg/m^2^ on days 5 and 36) was administered. Radiation was delivered using intensity-modulated radiotherapy (IMRT) at a daily dose of 1.7 to 2.0 Gy, with cumulative doses ranging from 60 to 70.4 Gy over 30–35 fractions across 6–8 weeks [2,20].

### 2.2. Statistical Analysis

For descriptive statistics, continuous variables, such as changes in body composition, caloric intake, and biochemical markers, were summarized using medians with corresponding ranges.

Mann–Whitney U tests were used for continuous variables. Chi-square or Fisher’s exact tests were applied for categorical variables. 

Based on previously published data [2], not only the pre- and posttherapeutic BMI but also indicators from BIA (PA, FFMI), nutritional medical scores (MUST, NRS-2002, and Nutriscore), laboratory chemical indicators of nutritional status (albumin, total protein count), and C-reactive protein (CRP) as an indicator of inflammatory status were compared using the Wilcoxon rank test. For each parameter, a distinction was made between male and female patients. Parameters that showed a relevant pre- to posttherapeutic difference (*p* < 0.05) were then examined for their influence on survival using the log-rank test (univariable survival analysis). Since for male HNC patients, several parameters from the univariable analysis showed a *p* < 0.05, we subsequently performed a multivariable Cox proportional hazards model for the male cohort. This approach allowed us to assess gender-specific predictors of survival, considering multiple covariates simultaneously. Parameters included in this multivariable model were (baseline) calorie deficit, BMI (end of therapy), (baseline) PA, FFMI (both time points), and (baseline) MUST.

Cut-off values were either defined by means of receiver-operating characteristics and Youden-index calculations (calorie deficit, PA), by commonly used reference values (BMI, FFMI, MUST, CRP, total protein count), or by previously published data (albumin levels [20]). Statistical significance for all calculations was set at *p* < 0.05. All analyses were conducted using SPSS (version 28, IBM Corp).

## 3. Results

This analysis included 61 HNC patients, of whom 17 (27.9%) were women and 44 (72.1%) were men. Median follow-up in the whole cohort was 22 (1–33) months. Three patients were lost to follow-up, as one was refused entry to Germany (during the coronavirus pandemic), a second patient did not want follow-up examinations, and a third patient has not undergone further BIA measurements. Differences between both genders were observed regarding Karnofsky performance status (*p* = 0.01), daily calorie intake (*p* = 0.04), phase angle (*p* = 0.003), and FFMI (*p* < 0.001). Table 1 summarizes all baseline patient characteristics.

### 3.1. Gender-Specific Nutritional and Metabolic Changes

When analyzing nutritional and metabolic changes among male and female HNC patients during (chemo)radiotherapy, we observed notable gender-specific differences: Male patients showed a significantly larger increase in calorie deficit from baseline to the end of therapy (median difference of −394 kcal, *p* < 0.001) compared to female patients, who had a smaller median difference of −164 kcal (*p* = 0.68).

In both genders, BMI decreased significantly, with males experiencing a greater reduction (median change of −1.2 kg/m^2^, *p* < 0.001) than females (−0.81 kg/m^2^, *p* = 0.02). Similarly, FFMI declined significantly in males (median change of −0.7 kg/m^2^, *p* < 0.001) compared to females (−0.3 kg/m^2^, *p* = 0.02). For PA, an indicator of cellular integrity, males experienced a significant decrease (median change of −0.6°, *p* = 0.04), while females showed a slight, non-significant increase (+0.4°, *p* = 0.72).

Scores from the MUST questionnaire indicated a significant increase in malnutrition risk for males (*p* = 0.008) but no substantial change for females (*p* = 0.09). Both groups exhibited similar median increases in Nutriscore.

Albumin levels decreased significantly in both groups, with a more pronounced reduction in females (median change of −5.6 g/L, *p* = 0.004) than males (−2.9 g/L, *p* < 0.001). Total protein count similarly declined in both genders, though the drop was slightly greater in females (−5.7 g/L vs. −3.1 g/L for males, both *p* < 0.001). Male patients showed a significant increase in CRP, indicating inflammation (median difference of +4 mg/L, *p* = 0.03), whereas CRP levels in females remained stable (*p* = 0.4). Table 2 summarized all pre- to posttherapeutic changes.

### 3.2. Gender-Specific Survival

For univariable analysis, all parameters showing relevant changes (*p* < 0.05) under (chemo)radiotherapy (Table 2) were subsequently tested for their gender-specific influence on survival (log-rank test).

For male patients, calorie deficit was significantly associated with survival at baseline (*p* = 0.04), indicating that patients with a baseline calorie deficit above the cut-off of 392.61 kcal had poorer survival outcomes. However, this association was weaker by the end of therapy (*p* = 0.08). In addition, male BMI at the end of therapy (*p* = 0.005), PA at both baseline (*p* = 0.002) and at the end of therapy (*p* = 0.05), FFMI at both time points (*p* < 0.001 each), and baseline MUST scores (*p* = 0.03) were significantly associated with survival. 

For female patients, only albumin values at the end of therapy (*p* < 0.001) seemed to be an indicator of survival. Table 3 summarizes all gender-specific results from univariable survival analysis.

Multivariable survival analysis revealed that for male patients, baseline PA was significantly associated with survival (*p* = 0.026), with a lower PA indicating poorer survival outcomes (Exp (B) = 0.118; 95% CI: 0.018–0.778). Other indicators, such as baseline calorie deficit, BMI at the end of therapy, and FFMI at both time points, were not significantly associated with survival for male patients.

## 4. Discussion

Based on the prospective HEADNUT patient cohort [2], this analysis provides insights into gender-specific nutritional and metabolic responses among HNC patients undergoing CRT. Moreover, it examines the influence of these factors on gender-specific survival. Our findings revealed several significant baseline differences between male and female patients, notably in functional status, daily calorie intake, PA, and FFMI, with men generally displaying higher FFMI and PA values. 

In 2023, Gaeta et al. analyzed the inclusion of sex/gender in HNC studies. They pointed out the necessity to include this feature in upcoming trials and to raise awareness of its importance [22]. Nevertheless, data on nutritional gender differences in HNC are very limited. Martínez-Herrera et al. examined the importance of PA on the gender-specific prognosis and quality of life of 139 HNC patients. A lower PA was associated with poorer quality of life. The gender-specific analysis revealed a pronounced inflammatory situation in the female sub-cohort [11]. In our cohort, the CRP value increased significantly at the end of therapy in both genders.

In line with the generally known prevalence of HNC in males [23,24], more men were affected in our analysis. Under ongoing therapy, male patients experienced more pronounced calorie deficits, reductions in BMI, and declines in both FFMI and PA compared to female patients. Per se, men have a higher daily energy requirement and achieve higher PA values in the BIA [11,25]. Swiss data recommend a higher cut-off value for FFMI in men (17 kg/m^2^) than in women (15 kg/m^2^) [26]. On the one hand, these reductions in key nutritional indicators might reflect a heightened vulnerability to muscle and cellular loss in male patients, which could have critical implications for their overall physical resilience and recovery. This would assume an increased malnutrition risk in males (as indicated by MUST scores), suggesting that men might be at a higher risk of therapy-induced nutritional deterioration. In contrast, female patients maintained more stable PA and calorie intake levels, possibly hinting at better cellular integrity or adaptive metabolic responses during treatment. On the other hand, psychological factors can also play a role: It can be assumed that women are more adherent to nutritional therapy or may consult medical staff earlier if they experience unwanted weight loss.

When examining inflammatory and protein markers, albumin levels and total protein count decreased significantly in both groups. However, this decline seemed to be more pronounced in females. Both genders showed a significant increase in CRP levels [11], indicating increasing levels of inflammation. Differences in inflammatory response among both genders may partly explain the gender-specific survival outcomes observed in our study. Male patients’ survival was strongly associated with calorie intake, BMI, PA, and FFMI, suggesting that maintaining nutritional and functional metrics is crucial for their prognosis. Per se, we would have expected the same effects in female HNC patients but were not able to prove this. Nevertheless, in the initial HEADNUT cohort, FFMI, albumin, and PA were indicators of OS [2]. Conversely, for female patients, only albumin levels at the end of therapy correlated with survival, emphasizing the importance of protein reserves as a potential survival indicator. In this study, we tried to highlight that while inflammatory responses differ between genders, the relationship between CRP levels and survival outcomes is complex and multifactorial. For women, survival outcomes were strongly linked to albumin levels at the end of therapy, which might reflect their ability to maintain protein reserves despite inflammation. From our perspective, chronic inflammation, as reflected by elevated CRP, is often associated with worse prognosis in cancer patients, as it can drive tumor progression, immunosuppression, and metabolic dysregulation. Nevertheless, we assume that other factors, such as baseline nutritional status or the ability to recover metabolically during treatment, may play a more significant role in influencing survival outcomes.

It is indisputable that our findings highlight the need for further gender-specific and personalized approaches. One could assume that especially male patients may benefit from targeted nutritional support to mitigate calorie deficits and muscle loss, potentially enhancing their overall prognosis. Conversely, ensuring adequate protein intake for female patients could help improve their therapeutic resilience and survival.

Although a sample size calculation was carried out before the initiation of the HEADNUT trial, it was primarily powered for malnutrition endpoints. Therefore, a major limitation of the present analysis is that it was not powered for the endpoint investigated here (gender-specific differences). The relatively small sample size, particularly the limited number of female patients, may reduce the statistical power and generalizability of our findings. Type I and Type II errors, potentially leading to false positives or overlooked significant effects, respectively, cannot be excluded. Another limitation is the reliance on self-reported data for calorie intake, which can be prone to bias, particularly if patients underreport or inaccurately recall their dietary intake. This could affect the accuracy of our calorie deficit calculations and potentially distort gender comparisons. Moreover, while we analyzed important nutritional and inflammatory biomarkers like albumin and CRP, other relevant biochemical markers were not included, which may have limited our understanding of the broader metabolic and inflammatory changes during treatment.

In our study, patients were included regardless of tumor stage and location, as the primary focus was on gender-specific differences in nutritional and biochemical responses. We acknowledge that tumor stage can significantly influence overall survival and baseline calorie deficits, particularly due to tumor-related cachexia. A more detailed analysis comparing relevant stages and specific tumor locations between genders would indeed be beneficial but lies beyond the scope of the current work. Nevertheless, we consider this an important aspect for future studies to further explore the interplay between tumor stage, location, and gender-specific differences. Interestingly, although there were no statistical differences between the locations of the tumor primaries between both genders. However, more men appeared to have suffered from oropharyngeal cancer. Since it could be assumed that there is a spatial proximity to the salivary glands and that it may not have been possible to protect them from radiotherapy, it cannot be ruled out that men were at higher risk of xerostomia and thus a deterioration in nutritional status. Regarding the type of therapy chosen, also no statistically significant differences between either gender could be demonstrated. However, more men (43.2%) received primary chemoradiotherapy. This could also be a possible explanation for the more expressed nutritional deterioration in men, as in this case more side effects are to be expected than with radiation alone.

## 5. Conclusions

Being predictive for OS, our findings highlight the importance of personalized nutritional monitoring during CRT. To be more specific, male patients may benefit from targeted nutritional support to mitigate calorie deficits and muscle loss, potentially enhancing their overall prognosis, and, conversely, ensuring adequate protein intake for female patients could help improve their therapeutic resilience and survival. However, our findings should be validated on a larger patient population.

## Figures and Tables

**Table 1:** Baseline gender-specific patient characteristics.

Features	Female (♀) HNC Patients		Male (♂) HNC Patients		
	Number	%	Number	%	*p*-Value
	*n* = 17	27.9	*n* = 44	72.1	
Age	71 (49–82)	-	61 (20–89)	-	0.08 ^1^
Karnofsky performance status (%)	80 (70–100)	-	90 (60–100)	-	**0.01 ^1^**
Primary tumor site					0.09 ^2^
(a) Oropharynx	8	47.1	30	68.2	
(b) Oral cavity	5	29.4	5	11.4	
(c) Hypopharynx	3	17.6	1	2.3	
(d) Larynx	1	5.9	4	9.1	
(e) Other (e.g., multistage)	-	-	4	9.1	
HPV status					0.42 ^2^
Positive	7	41.2	26	59.1	
Negative	6	35.3	12	27.3	
N/a	4	23.5	6	13.6	
UICC stage (version 8)					0.92 ^2^
I	5	29.4	14	31.8	
II	3	17.6	8	18.2	
III	4	23.5	7	15.9	
IV	5	29.4	15	34.1	
T-stage					0.13 ^2^
T1	2	11.8	7	15.9	
T2	6	35.3	18	40.9	
T3	5	29.4	3	6.8	
T4	4	23.5	16	36.4	
N-stage					0.14 ^2^
N0	5	29.4	6	13.6	
N1	8	47.1	17	38.6	
N2	2	11.8	18	40.9	
N3	2	11.8	3	6.8	
Treatment mode					0.54 ^2^
(a) Primary					
Concurrent chemotherapy	4	23.5	19	43.2	
Radiotherapy alone	2	11.8	4	9.1	
				
(b) Adjuvant					
Concurrent chemotherapy	4	23.5	9	20.5	
Radiotherapy alone	7	41.2	12	27.3	
Concurrent chemotherapy (as initially prescribed):					0.52 ^2^
Cisplatin 100 mg/m^2^ q3w	5	29.4	10	22.7	
Cisplatin 40 mg/m^2^ weekly	3	17.6	15	34.1	
5-FU/Mitomycin C	-	-	2	4.5	
Cetuximab	-	-	1	2.3	
None	9	52.9	16	36.4	
Reached cumulative cisplatin dose					0.65 ^3^
<200 mg/m^2^	1	5.9	6	13.6	
≥200 mg/m^2^	7	41.2	19	43.2	
Cumulative RT dose (Gy)	66 (60–70)	-	66 (60–70.4)		0.17 ^1^
Smoking status					0.53 ^2^
Never smoked	5	29.4	16	36.4	
Active smoker	3	17.6	12	27.3	
Ex-smoker	3	17.6	8	18.2	
Unknown	6	35.3	8	18.2	
Consumption of alcohol					0.51 ^2^
No alcohol	2	11.8	9	20.5	
Continued alcohol consumption	8	47.1	18	40.9	
Formerly consumed alcohol	1	5.9	7	15.9	
Unknown	6	35.3	10	22.7	
BMI (kg/m^2^)	22 (18.8–34.9)	-	24.6 (14.5–37.2)	-	0.23 ^1^
Daily calorie intake (kcal)	1758.3 (464.1–3808.1)	-	2306.2 (1161.1–4441)	-	**0.04 ^1^**
Phase angle before (°)	4.8 (3.5–5.9)	-	5.7 (3.7–7.7)	-	**0.003 ^1^**
FFMI (baseline)	15.8 (14.1–19.1)	-	18.9 (14–24.7)	-	**<0.001 ^1^**

Patient-specific gender baseline characteristics. HNC = head and neck cancer, HPV = human papilloma virus, N/a = not available, RT = radiotherapy, BMI = body mass index, FFMI = fat-free mass index, UICC = Union Internationale Contre le Cancer, 5-FU = 5-Fluoruracil. ^1^ Mann–Whitney U test. ^2^ Chi-square test. ^3^ Fisher’s exact test. *p*-values <0.05 were marked in bold.

**Table 2:** Summary of pre- to posttherapeutic changes in nutritional indicators.

	Female HNC Patients				Male HNC Patients			
	Baseline	End of Therapy	*p*-Value	Median Absolute Difference (Δ)	Baseline	End of Therapy	*p*-Value	Median Absolute Difference (Δ)
Calorie deficit (kcal)	239.2 (−1355.7–2337.9)	−83.5 (−1269.1–4727.9)	0.68	−164	99.2 (−1098.9–2179.2)	−557 (−2082.7–1523.8)	**<0.001**	−394
**Anthropometric nutritional indicators**								
BMI (kg/m^2^)	22 (18.8–34.9)	21.6 (17.8–32.8)	**0.02**	−0.81	24.6 (14.5–37.2)	22.8 (16.8–33)	**<0.001**	−1.2
PA (°)	4.8 (3.5–5.9)	4.9 (2.8–6.1)	0.72	+0.4	5.7 (3.7–7.7)	5 (3.1–7.1)	**0.04**	−0.6
FFMI (kg/m^2^)	15.8 (14.1–19.1)	15.5 (13.4–19.2)	**0.02**	−0.3	18.9 (14–24.7)	18.3 (14.4–21.6)	**<0.001**	−0.7
**Nutritional screening questionnaires**								
MUST	1 (0–4)	1 (0–4)	0.09	+1	1 (0–5)	2 (0–5)	**0.008**	+1
NRS-2002	3 (0–5)	3 (0–4)	0.29	0	2 (0–4)	3 (0–4)	0.4	0
Nutriscore	4 (0–4)	6 (3–8)	0.33	+2	4 (2–7)	6 (3–8)	0.46	+2
**Laboratory parameters**								
Albumin (g/L)	36.3 (28.3–42)	33.4 (21–38.4)	**0.008**	−7.2	35.5 (24.9–44.1)	31.4 (20.4–45.5)	**<0.001**	−3.4
CRP (mg/L)	5 (0–54)	22 (0–71)	**0.04**	+13	0 (0–125)	8 (0–74)	**0.003**	0
Total protein count (g/L)	72.4 (63–86.2)	64.2 (54–77.7)	**0.003**	−7	73.6 (64–86.1)	69.4 (50.3–84.4)	**<0.001**	−4

Gender-specific pre- to posttherapeutic changes. Calorie deficit refers to the difference between the actual calorie intake and the calculated, necessary calorie intake to maintain the current body weight. HNC = head and neck cancer, PA = phase angle, BMI = body mass index, FFMI = fat-free mass index, CRP = C-reactive protein, MUST = Malnutrition Universal Screening Tool, NRS-2002 = Nutritional Risk Screening 2002. *p*-values <0.05 were marked in bold.

**Table 3:** Univariable survival analysis of nutritional indicators.

	HNC Patients	
	Female (♀)	Male (♂)
	Log-Rank *p*	Log-Rank *p*
Calorie deficit (kcal)		
Baseline (cut-off: 392.6 ^1^)	0.29	0.04
End of therapy (cut-off: −62.34 ^1^)	0.38	0.08
BMI (kg/m^2^) (cut-off: 18.5 ^2^)		
Baseline	-	0.15
End of therapy	0.72	0.005
PA (°) (cut-off: 5.6 ^1^)		
Baseline	0.72	0.002
End of therapy	0.58	0.05
FFMI (kg/m^2^) (cut-off: <15 (♀) and <17 (♂ ^2^))		
Baseline	0.52	<0.001
End of therapy	0.52	<0.001
MUST (cut-off: <2 vs. ≥2 ^2^)		
Baseline	0.52	0.03
End of therapy	0.35	0.27
Albumin (g/L) (cut-off: 37.5 ^3^)		
Baseline	0.46	0.15
End of therapy	<0.001	0.48
CRP (mg/L) (cut-off: 5 ^2^)		
Baseline	0.29	0.74
End of therapy	0.07	0.27
Total protein count (g/L) (cut-off: 66 ^2^)		
Baseline	0.64	0.41
End of therapy	0.29	0.24

Gender-specific univariable survival analysis. Calorie deficit refers to the difference between the actual calorie intake and the calculated, necessary calorie intake to maintain the current body weight. HNC = head and neck cancer, PA = phase angle, BMI = body mass index, FFMI = fat-free mass index, CRP = C-reactive protein, MUST = Malnutrition Universal Screening Tool. ^1^ Youden index. ^2^ Reference value. ^3^ Recommended cut-off value according to León et al. [21].

## Data Availability

Data are unavailable due to privacy.
